# Immunization against covid-19 and mortality in hospitalized patients: a retrospective cohort

**DOI:** 10.11606/s1518-8787.2024058005476

**Published:** 2024-03-04

**Authors:** Alexandre Medeiros de Figueiredo, Adriano Massuda, Michelle Fernandez, Agostinho Hermes de Medeiros, Marcus Carvalho

**Affiliations:** I Universidade Federal da Paraíba Centro de Ciências Médicas Departamento de Promoção da Saúde João Pessoa PB Brasil Universidade Federal da Paraíba. Centro de Ciências Médicas. Departamento de Promoção da Saúde. João Pessoa, PB, Brasil; II Universidade Federal da Paraíba Departamento de Estatística Programa de Pós-Graduação em Modelos de Decisão em Saúde João Pessoa PB Brasil Universidade Federal da Paraíba. Departamento de Estatística. Programa de Pós-Graduação em Modelos de Decisão em Saúde. João Pessoa, PB, Brasil; III Fundação Getulio Vargas Escola de Administração de Empresas do Estado de São Paulo Centro de Estudos de Planejamento e Gestão em Saúde São Paulo SP Brasil Fundação Getulio Vargas. Escola de Administração de Empresas do Estado de São Paulo. Centro de Estudos de Planejamento e Gestão em Saúde (FGV-Saúde). São Paulo, SP, Brasil; IV Universidade de Brasília Instituto de Ciência Política Brasília DF Brasil Universidade de Brasília. Instituto de Ciência Política. Brasília, DF, Brasil; V Universidade Federal da Paraíba Centro de Ciências Médicas Departamento de Doenças Infecciosas, Parasitárias e Inflamatórias João Pessoa PB Brasil Universidade Federal da Paraíba. Centro de Ciências Médicas. Departamento de Doenças Infecciosas, Parasitárias e Inflamatórias. João Pessoa, PB, Brasil; VI Universidade Federal da Paraíba Centro de Ciências Aplicadas e Educação Departamento de Ciências Exatas Rio Tinto PB Brasil Universidade Federal da Paraíba. Centro de Ciências Aplicadas e Educação. Departamento de Ciências Exatas. Rio Tinto, PB, Brasil

**Keywords:** Covid-19, Covid-19 Vaccines, Mortality, Observational Study, Cohort Studies

## Abstract

**OBJECTIVE:**

To evaluate the effectiveness of vaccines developed against covid-19 in reducing mortality in people hospitalized with severe acute respiratory syndrome (SARS) caused by SARS-CoV-2.

**METHODS:**

This is a retrospective cohort that evaluated risk factors and the effectiveness of the two-dose vaccination schedule in reducing the mortality of people hospitalized for covid-19 in the state of Paraíba from February to November 2021. The explanatory variables were vaccination status, presence of comorbidities, socioeconomic and demographic characteristics. Descriptive analyses and bivariate and multivariable logistic regression were performed.

**RESULTS:**

Most hospitalizations and deaths occurred until May 2021. The percentage of patients with a complete vaccination schedule was similar across patients admitted to public and private hospitals and higher in residents of less developed municipalities. Multivariable analysis demonstrated that women (OR = 0.896; 95%CI 0.830–0.967) and people admitted to private hospitals (OR = 0.756; 95%CI 0.679–0.842) were less likely to die. Presence of any comorbidity (OR = 1.627; 95%CI 1.500–1.765) and age ≥ 80 years (OR = 7.426; 95%CI 6.309–8.741) were risk factors for death. Patients with complete vaccination schedule at the time of admission were 41.7% less likely to die (OR = 0.583; 95% CI 0.501–0.679) from covid-19 in the adjusted analysis, as compared to unvaccinated patients.

**CONCLUSIONS:**

The study reveals that immunization was effective in reducing the likelihood of death from covid-19. The results suggest that greater vaccination coverage in the first half of 2021 would prevent thousands of deaths in the country.

## INTRODUCTION

Covid-19 is an infectious disease caused by the new severe acute respiratory syndrome coronavirus 2 (SARS-CoV-2), which emerged at the end of 2019. By the end of 2022, the covid-19 pandemic had caused the death of 6.67 million people worldwide^1^. Of this total, 694,000 (10.4%) occurred in Brazil^2^.

Around the world, the severity of the covid-19 pandemic led countries to adopt measures to contain the spread of the health crisis. Among the strategies adopted, immunization against covid-19 has successfully protected the population and helped confront the health crisis^3,4^. After the development of different types of vaccines, the high rate of vaccination coverage was the most important strategy to face the pandemic^5^, combined with other non-pharmacological measures to control contagion^6^.

Efforts to develop vaccines began in March 2020. During the first year of the pandemic, several laboratories disclosed their vaccine development processes. These processes were closely monitored by the international community^9,10^. In Brazil, vaccination of the population began in January 2021, after the approval of the Coronavac immunizers (live attenuated virus) from Sinovac/Butantã and the ChAdOx1 nCoV-19 immunizer (recombinant) produced by the AstraZeneca/Fiocruz partnership. Subsequently, the Pfizer–BioNTech vaccine (RNA Messenger) and the vaccine produced by the pharmaceutical company Janssen (recombinant) were incorporated into the National Immunization Program (PNI)^11^.

The vaccines used in Brazil are highly effective in preventing moderate and severe cases, which can be observed in clinical trials^12^and effectiveness studies in other countries^15^. In effectiveness studies, the data indicate that immunizers protect against progression to severe forms, with a reduction in mortality from covid-19 , which was 86.3% for the Coronavac immunizer and 96.7% for the Pfizer–BioNTech immunizer. Likewise, a reduction of at least 85% in hospitalizations due to covid-19 was demonstrated^15^.

Despite the evidence of vaccine effectiveness in the general population, little is known about how vaccination status influences mortality in the subgroup of patients who progress to severe forms of the disease. It is noteworthy that there are few studies on the effectiveness of vaccines in developing countries. The authors’ hypothesis is that the development of active immunity from covid-19 vaccines provides individuals with a better immune response during severe forms of the disease. In this sense, this study aimed to evaluate the effectiveness of the complete vaccination schedule in reducing the mortality of people hospitalized due to severe acute respiratory syndrome (SARS). In addition, we assessed the risk factors for mortality from covid-19.

## METHODS

This is a retrospective cohort study, based on data extracted from the Influenza Epidemiological Surveillance Information System (SIVEP-Gripe) database and the national base of the National Immunization Program (SIS-PNI), referring to 2021, made available by the State Department of Health of Paraíba (SES-PB) and updated until November 6, 2021. The choice for the state of Paraíba was for convenience, due to the partnership with the Federal University of Paraíba (UFPB) and access to hospitalization and immunization data.

The state of Paraíba has 223 municipalities and a high Primary Care coverage, presenting the fourth highest Family Health Strategy coverage in the country in 2019 (86.7%)^18^. The hospital bed ratio is 205.3 beds per 100,000 inhabitants, slightly higher than the national average^19^. The accumulated incidence of COVID-19 in the state was 17,625.2 cases per 100,000 inhabitants in mid-March 2023, close to the national average. Mortality from covid covid-19, in turn, was 262.3 deaths per 100,000 inhabitants, while the national average was 332.9 deaths per 100,000 inhabitants^2^.

The study included people aged ≥ 18 years who were hospitalized with COVID-19 and its first clinical symptoms from February 1, 2021 to November 6, 2021. People with COVID-19 were considered to be all those who met the Ministry of Health’s diagnostic criteria (laboratory confirmation, clinical epidemiological criteria) classified in SIVEP-Gripe as severe acute respiratory syndrome due to covid-19 , and who were registered as hospitalized.

Cases in which the hospitalization record and clinical evolution (death from covid-19 , death from other causes or cure) were not recorded in the SIVEP-Gripe database were excluded from the study. The authors chose to evaluate data after the start of the immunization process in order to ensure that immunized and non-immunized people were exposed to similar viral strains.

Patient identification information, sex, date of first symptoms, age, presence of risk factors for covid-19 (presence or absence of any risk factor), code of the hospitalization establishment in the National Registry of Establishments (CNES), municipality of residence, date of hospitalization and evolution of the case were extracted from the SIVEP-Gripe database. Information regarding type of immunizer, date of vaccine administration, vaccine dose and identification data of the immunized person were extracted from the SIS-PNI. An initial cleaning of the SIS-PNI data was carried out to standardize records of the same type of immunizer that were recorded in different ways and to remove duplicate records of vaccine doses for the same patient. Then, the SIS-PNI and SIVEP-Gripe databases were aggregated using the deterministic record linkage technique, using the *Cadastro de Pessoa Física* (Individual Taxpayer Registry - CPF) as the key. In records where this information was missing, a second linkage was carried out*,* using the patient’s name and date of birth variables. Data filtering, cleaning and joining processes were carried out using the R language with the tidyverse^20^ package.

After aggregating the databases, the dataset was manually reviewed to identify duplicate notifications and notifications in different facilities during the clinical evolution of the same SAR-CoV-2 infection, as some patients were admitted to more than one facility (transfer or readmission). In these cases, the notification with the most recent evolution date was considered. In cases where the difference between hospitalization dates was greater than 60 days, a new infection was considered and the record was not excluded.

The clinical outcome assessed was death from covid-19 and the explanatory variables were sex, race/color, type of hospital (public or private), Municipal Human Development Index (MHDI) of the municipality of residence, patient age, presence of comorbidity, and vaccination status at the date of onset of the first symptoms. The race/color classification followed the categorization of the Brazilian Institute of Geography and Statistics (IBGE). People categorized as Mixed-race and Black were grouped into the Black category. Public hospitals were considered to be those under public management; private hospitals were considered to be those for-profit and philanthropic, using the legal classification of the CNES database as a parameter. The municipalities were categorized using the cuttoff points on the HDI proposed by United Nations: low HDI < 0.555; medium from 0.555 to 0.699; high from 0.700 to 0.799, and very high greater than or equal to 0.800^21^. The vaccination status was defined based on the time elapsed between the date of administration of the vaccine doses and the date of the first symptoms. All patients without a dose of vaccine or with an administration carried out 14 days before the date of onset of the first symptoms were considered unvaccinated. Patients with a time interval greater than 14 days between the date of the second dose of the vaccine and the date of the first symptoms were considered completely immunized and those in whom only one dose of the vaccine was administered at least 14 days before the first symptoms were considered partially immunized.

The study population was described in subgroups categorized based on the characteristics referring to the explanatory variables, using absolute values and the proportion of each subgroup in relation to the total number of individuals studied. Logistic regression was performed and odds ratio (OR) values and their respective confidence intervals (95% CI) were calculated for each of the factors analyzed, using the bivariate logistic regression method and adjusted OR values and their confidence intervals (95% CI), based on multivariable analysis. The race/color variable was not evaluated in the regression models due to the high proportion of missing data, especially in records from private hospitals.

Additionally, monthly vaccination coverage data for the first and second doses of vaccines against covid-19 in the population of Paraíba were calculated using data from the SIS-PNI and data referring to the population of Paraíba by age group.

In compliance with the recommendations contained in Resolution No. 466/2012, of the National Health Council, the project of this study was submitted to and approved by the Ethics Committee for Research with Human Beings, of the UFPB Centro de Ciências Médicas, under CAAE No. 50833321.4.0000.8069.

## RESULTS

**Figure 1:**
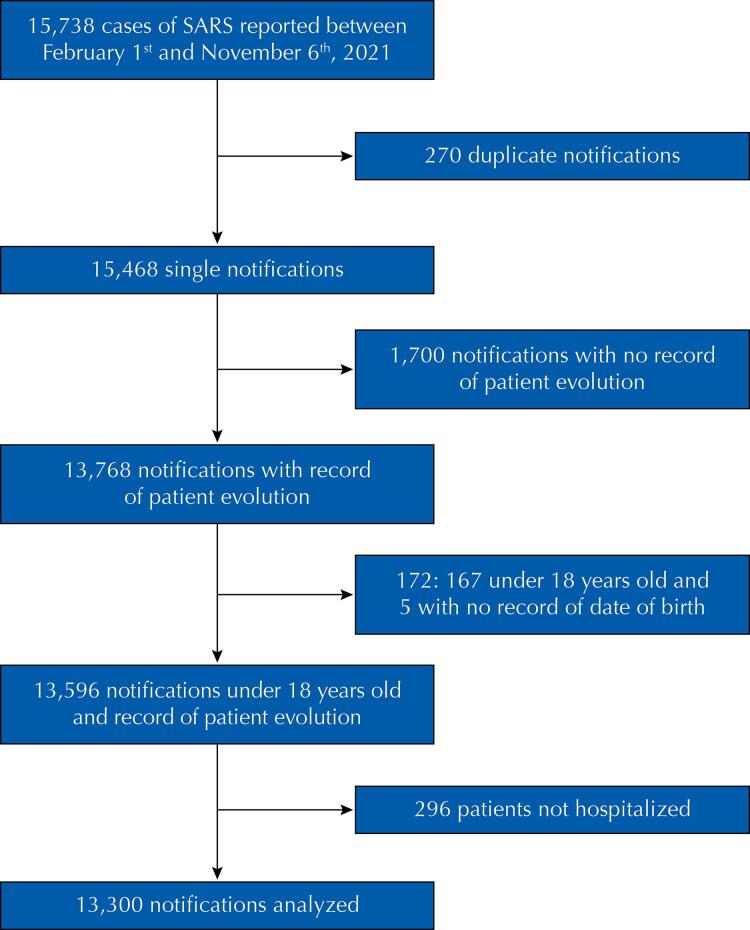
Flowchart of participant inclusion from SARS notifications according to the study’s inclusion and exclusion criteria. SARS: severe acute respiratory syndrome.

During the studied period, there were 15,738 records of SARS cases attributed to infection with SAR-CoV-2. Cleaning the database and analyzing whether the inclusion criteria were met resulted in a database with 13,300 hospitalizations (Figure 1).

Of the hospitalizations analyzed, 76% occurred from February to May 2021. Most people hospitalized were men (55.95%), had comorbidities (58.27%), were Black (79.19%) and were admitted to public hospitals (80.35%) (Table 1). The race/color variable presented the worst completion across the variables evaluated. The percentage of patients without race/color recorded in notifications was 2.40% in public hospitals and 55.34% in private hospitals. The percentage of Black patients admitted to hospitals ranged from 52.64% in private hospitals to 93.04% in public hospitals. Among patients who died, 74.26% had access to Intensive Care Units (ICU) in public hospitals and 84.78% in private hospitals. Most hospitalized patients were aged ≤ 60 years (59.64%) and the average age was 56.64 years. The lethality of SARS cases due to COVID-19 in hospitalized patients was 36.22%, varying by age group, being 19.13% in patients up to 39 years old and 60.73% in patients aged ≥ 80 years old. Lethality varied over time, being 41.1% in February and March and 29.6% in June and July.

**Table 1:** Characteristics of patients hospitalized for SARS in the state of Paraíba, according to vaccination status before covid-19 infection.

Characteristic	Vaccination status prior to the date of first symptoms							
	Complete schedule		Incomplete schedule		No vaccine		Total	
	n	(%)	n	(%)	n	(%)	n	(%)
Sex								
Male	495	49.25	738	52.05	6,209	57.08	7,442	55.95
Female	510	50.75	680	47.95	4,668	42.92	5,858	44.05
Race/color								
White	110	10.17	122	11.28	850	78.56	1,082	8.14
Black	770	7.31	1,128	10.71	8,634	81.89	10,532	79.19
Yellow	10	7.81	9	7.03	109	85.16	128	0.96
Indigenous	3	20	two	13.33	10	66.67	15	0.11
No information	112	7.26	157	10.17	1,274	82.57	1,543	11.6
Presence of risk factor								
No	235	23.38	381	26.87	4,934	45.36	5,550	41.73
Yes	770	76.62	1,037	73.13	5,943	54.64	7,750	58.27
Age range (years)								
18–39	18	1.79	59	4.16	2,427	22.31	2,504	18.83
40–49	17	1.69	65	4.58	2,469	22.7	2,551	19.18
50–59	29	2.89	115	8.11	2,733	25.13	2,877	21.63
60–69	133	13.23	420	29.62	1,557	14.31	2,110	15.86
70–79	388	36.61	334	23.55	1,054	9.69	1,776	13.35
≥ 80	420	41.79	425	29.97	637	5.86	1,482	11.14
Type of hospital								
Public	825	7.72	1,143	10.7	8,719	81.59	10,687	80.35
Private	151	6.72	238	10.6	1,857	82.68	2,246	16.89
No information	29	7.9	37	10.08	301	82.02	367	2.76
HDI of the municipality of residence								
Low	44	9.61	61	13.32	353	77.07	458	3.44
Medium	565	9.02	654	10.44	5,048	80.55	6,267	47.12
High	395	6.04	698	10.67	5,451	83.3	6,544	49.2
Very high	1	3.23	55	16.13	25	80.6	81	0.23
Type of immunizer								
Sinovac/Butantã	936	7.04	774	5.82	-	-	1,710	12.86
Oxford/AstraZeneca/Fiocruz	69	0.52	588	4.42	-	-	657	4.94
Janssen Cilag	-	-	7		-	-	7	0.05
Pfizer BioNTech	-	-	48		-	-	48	0.36

HDI: Human Development Index.

As seen in Table 1, the group of people hospitalized with a complete vaccination schedule presented different characteristics from the group without immunization prior to hospitalization for SARS, being predominantly composed of older adults (93.63%) and people with risk factors for death from covid-19 (76.62%). The percentage of Indigenous individuals with a complete vaccination schedule was higher than that of the other groups, as a result of being a priority group by the PNI. Patients admitted to hospitals with a complete vaccination schedule had been administered with vaccines from Sinovac/Butantã (93.13%) and Oxford/AstraZeneca/Fiocruz (6.87%). These immunizers were also the most administered among patients with an incomplete vaccination schedule. The percentage of patients with a complete vaccination schedule was similar across patients hospitalized in public and private facilities. In hospitalized people, the median time elapsed between the second dose of the vaccine and the date of the first symptoms was 79 days (interquartile range 46 to 124 days). It is noteworthy that the highest percentage of hospitalized patients with a complete vaccination schedule occurred in municipalities with low and medium MHDI. Heart disease, diabetes and obesity were the most common risk factors in hospitalized patients (Table 2).

**Table 2:** Prevalence of risk factors in hospitalized patients.

Risk factor	Prevalence (%)
Heart disease	27.36
Diabetes	9.76
Obesity	11.98
Immunosuppression	1.45
Lung disease	1.91
Asthma	1.86
Liver disease	0.56
Renal	2.37
Postpartum	0.5
Down’s syndrome	0.44
Hematological	0.34
Other comorbidities	34.39

**Figure 2:**
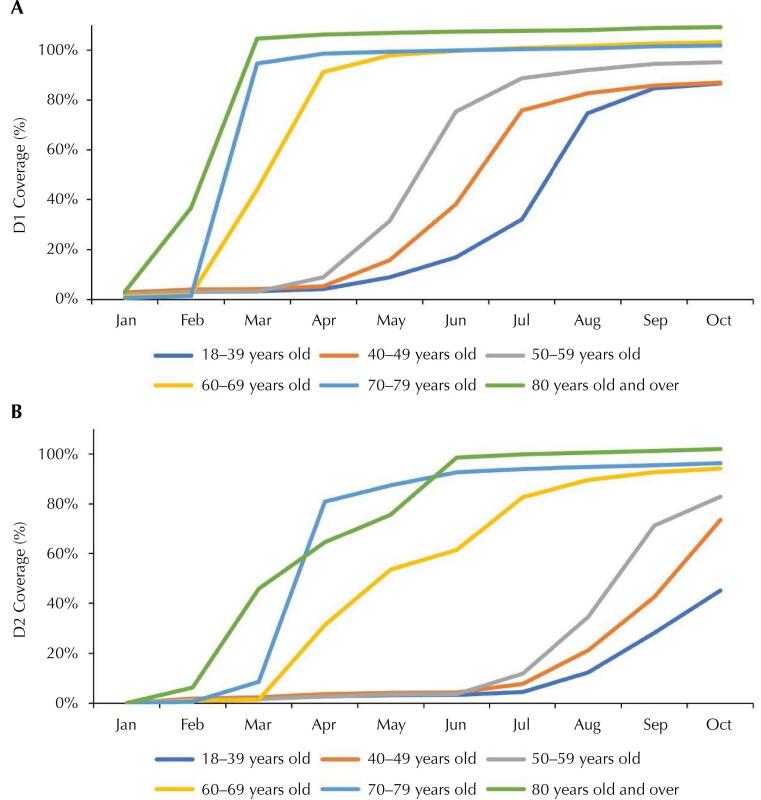
Vaccination coverage for the first (2A) and second dose (2B) of the vaccine against covid-19 in the adult and elderly population in the state of Paraíba, by age group.

Vaccination coverage in Paraíba varied by age group, with lower coverage for younger people (Figure 2). The population over 60 years of age had coverage greater than 90% in the first dose from April onwards. The reach of the second dose was lower than that observed in the first, with percentages above 90% identified only in the population aged 70 or over.

**Figure 3:**
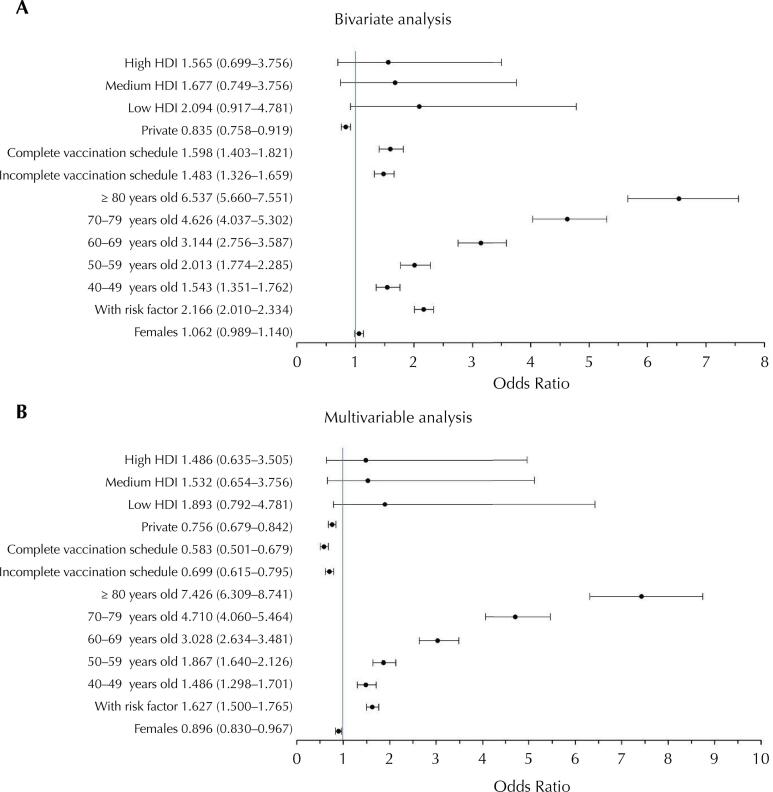
Odds ratio for progress to death in patients hospitalized with severe acute respiratory syndrome. Bivariate (Figure 3A) and multivariable (Figure 3B) analyses, considering biological characteristics, vaccination status, and care and socioeconomic characteristics.

The data from the multivariable analysis, described in Figure 3, demonstrate that women are less likely to die than men (OR = 0.896; 95%CI 0.830–0.967). On the other hand, patients with some risk factor are 62.7% more likely to die than patients without risk factors (OR = 1.627; 95%CI 1.500–1.765). Age was another aspect associated with the progressive increase in the risk of death. The population aged ≥ 80 years was more likely to die (OR = 7.426; 95%CI 6.309–8.741) than the population from 18 to 39 years. Patients with a complete vaccination schedule were 41.7% less likely to die (OR = 0.583; 95%CI 0.501–0.679) from covid-19 compared to unvaccinated patients. Individuals with an incomplete vaccination schedule showed a reduced likelihood of death (OR = 0.699; 95%CI 0.615–0.795), but lower than the subgroup with a complete schedule. People admitted to private hospitals were less likely to die as compared to those admitted to public hospitals (OR = 0.756; 95%CI 0.679–0.842).

## DISCUSSION

Results indicate that the vaccines effectively reduced the likelihood of death from COVID-19 in people with severe forms of the disease. Some characteristics were associated with death from covid-19 , such as: male sex, age ≥ 40 years, presence of comorbidities and health conditions identified as risk factors in SIVEP-gripe and hospitalization in public hospitals.

Most hospitalizations analyzed occurred from February to May 2021, a period in which there was a shortage of vaccines in Brazil. At the end of May 2021, vaccination coverage of the second dose in Paraíba for people aged over 18 years was only 13.44%^22^. Despite the history of successful vaccination campaigns and a good acceptance rate for the COVID-19 vaccine, Brazil showed a low increase in vaccination coverage in the first half of 2021^23^. The delay in acquiring immunization agents, due to rejection of Pfizer’s proposals in 2020, meant that the country began immunization almost a month later than countries like Canada^23^. The smaller stock of immunizers also led to partial interruptions in the immunization process. A study using SIR (susceptible-infected-recovered) modeling points out that bringing forward the start of the vaccine and a higher rate of vaccination in the initial months could have prevented thousands of deaths ^23^.

A cohort study, which also used the SIVEP-Gripe database, analyzed 254,000 hospitalizations in Brazil in 2020 and found that 56% of hospitalized patients were male, 53% were aged ≥ 60 years and 84% had at least one comorbidity ^24^. In the Northeast region, the percentages were, respectively, 58% and 83% ^24^.

The high percentage of admissions to public hospitals reveals the importance of the Brazilian Unified Health System (SUS) in responding to the covid-19 epidemic. Data from patients hospitalized in Paraíba from February to November 2021 demonstrate a lower percentage of hospitalized people aged ≥ 60 years (40.4%) and had comorbidities (58.27%) as compared to SIVEP-Gripe data for the country and the Northeast region in 2020^24^. These population groups began the immunization process as a priority and had a higher percentage of individuals with a complete vaccination schedule as compared to younger patients or those without comorbidities. Thus, this reduction in the percentage of hospitalized patients indicates the impact of vaccination on the reduction of severe forms. The lethality found in Paraíba in 2021 (36.22%) was lower than that observed in the Northeast region in 2020 (48%)^24^. Similarly, the expansion of vaccination coverage among older Brazilian adults until mid-May 2021 was associated with declines in relative lethality compared to younger individuals^3^.

Most patients hospitalized for SARS during the study period had risk factors and were over 50 years old, which is in line with other studies^25,26^. The highest percentage of Black people hospitalized was similar to the one found for the Northeast in a previous study^24^. However, this percentage is higher than expected, as the Black population in the state of Paraíba represented 67.1% of the total in 2021^27^. This fact may indicate that Black people were more exposed to covid-19 contamination, according to other studies in the literature^28,29^. Another factor that may have contributed to this difference was the rate of non-completion of this variable in private facilities, where the percentage of White patients was higher, leading to an underestimation. This high percentage of non-completion highlights the importance of strengthening the process of team qualification and filling out this information in private establishments.

Another highlight is a 24.40% lower likelihood of death in people admitted to private hospitals. One of the hypotheses for the higher mortality in public hospitals is the lack of access to intensive care. The percentage of people who died without access to the ICU in public hospitals (25.7%) was 69% higher than that found in private hospitals (15.20%). International studies indicate that endemic inequalities in the burden of chronic diseases in people with socioeconomic vulnerabilities can contribute to higher mortality^28,29^. Thus, the impact of the social determination of diseases reflects pre-existing inequities, resulting in a covid-19 syndemic^30^. From an opposite perspective, the similarity across the percentages of patients with a complete immunization admitted to public and private hospitals and a higher percentage of people with two doses in municipalities with low and medium HDI among hospitalized people reveals that the PNI allowed access to the vaccine to people from all social classes and municipalities of all levels of human development, acting as a producer of equity.

The data indicate that immunization against COVID-19 acts as a protective factor in hospitalized patients. A clinical trial that evaluated the use of dexamethasone in patients hospitalized for covid-19 demonstrated a relative risk reduction of 17%^31^. Two clinical trials in hospitalized patients did not demonstrate a reduction in mortality with remdesivir^32,33^. The reduction in lethality identified in this retrospective cohort cannot be directly compared to the aforementioned clinical trials; however, it suggests that the role of immunization agents in protecting patients with severe forms of covid-19 is clinically relevant.

This study has limitations related to the use of secondary databases. The first limitation refers to patients whose diagnostic classification was not recorded. These cases may reflect underreporting in the number of people hospitalized for covid-19. Another limitation refers to cases of patients with COVID-19 whose death was attributed to another cause (25 patients). Despite the linkage strategies used, the identification of the hospitalized patients’ vaccination status may have been hampered due to missing data in the databases used or filling errors. The percentage of data on race/color in more than half of the notifications occurring in private hospitals stands out as a limitation. The findings refer to a specific moment in the epidemic when only a portion of the population was vaccinated. More than 93% of people who completed the full course used the Sinovac/Butantã vaccine. Thus, the results found are mostly related to the use of this immunizer. Sinovac/Butantã has the least effectiveness in reducing hospitalizations and deaths among the immunization agents used in Brazil ^34^. Other vaccines may generate a greater reduction in mortality. Therefore, further studies should be carried out to evaluate the reduction in the likelihood of death across all immunizers.

Other research has highlighted the importance of immunizations for reducing severe forms and mortality in the general population. This study concludes that people hospitalized with complete vaccination have lower lethality as compared to those who did not have the full schedule before hospitalization, providing further evidence of the importance of vaccines against covid-19. In addition to reducing morbidity and mortality, the PNI demonstrated, once again, its importance as an inducer of equity. There is the great challenge of expanding vaccination coverage in groups that have become refractory to the use of vaccines and that are still vulnerable to infection by SARS-CoV-2. Strengthening the SUS and PNI will also be necessary to reverse the low vaccination coverage of several vaccines.
