# Supplementary material for: Immunization against covid-19 and mortality in hospitalized patients: a retrospective cohort
Source: Rev Saude Publica. 2024 Mar 4;58:07. doi: 10.11606/s1518-8787.2024058005476 (PMC10926983; doi:10.11606/s1518-8787.2024058005476)
Supplement: Translation

# Translation: Imunização contra covid-19 e mortalidade em pacientes hospitalizados: coorte retrospectiva

**Authors:** Alexandre Medeiros de Figueiredo, Adriano Massuda, Michelle Fernandez, Agostinho Hermes de Medeiros, Marcus Carvalho

**Affiliations:** I Brasil Universidade Federal da Paraíba. Centro de Ciências Médicas. Departamento de Promoção da Saúde. João Pessoa, PB, Brasil; II Brasil Universidade Federal da Paraíba. Departamento de Estatística. Programa de Pós-Graduação em Modelos de Decisão em Saúde. João Pessoa, PB, Brasil; III Brasil Fundação Getulio Vargas. Escola de Administração de Empresas do Estado de São Paulo. Centro de Estudos de Planejamento e Gestão em Saúde (FGV-Saúde). São Paulo, SP, Brasil; IV Brasil Universidade de Brasília. Instituto de Ciência Política. Brasília, DF, Brasil; V Brasil Universidade Federal da Paraíba. Centro de Ciências Médicas. Departamento de Doenças Infecciosas, Parasitárias e Inflamatórias. João Pessoa, PB, Brasil; VI Brasil Universidade Federal da Paraíba. Centro de Ciências Aplicadas e Educação. Departamento de Ciências Exatas. Rio Tinto, PB, Brasil

**Keywords:** Covid-19, Vacinas contra Covid-19, Mortalidade, Estudos Observacional, Estudos de Coorte

## Abstract

**OBJETIVO:**

Avaliar a efetividade das vacinas desenvolvidas contra a covid-19 na redução da mortalidade em pessoas internadas com síndrome respiratória aguda grave (SRAG) causada pelo SARS-CoV-2.

**MÉTODOS:**

Trata-se de uma coorte retrospectiva que avaliou fatores de riscos e a efetividade do esquema vacinal com duas doses na redução da mortalidade de pessoas internadas por covid-19 no estado da Paraíba entre fevereiro e novembro de 2021. As variáveis explicativas foram situação vacinal, presença de comorbidades, características socioeconômicas e demográficas. Foram realizadas análises descritivas e regressão logística bivariada e multivariável.

**RESULTADOS:**

A maior parte das internações e óbitos ocorreram até maio de 2021. O percentual de pacientes com esquema vacinal completo foi similar entre pacientes internados em hospitais públicos e privados e superior em residentes de municípios com menor desenvolvimento. A análise multivariável demonstrou que mulheres (OR = 0,896; IC95% 0,830–0,967) e pessoas internadas em hospitais privados (OR = 0,756; IC95% 0,679–0,842) apresentaram menor chance de morte. A presença de alguma comorbidade (OR = 1,627; IC95% 1,500–1,765) e idade ≥ 80 anos (OR = 7,426; IC95% 6,309–8,741) foram fatores de risco de óbito. Pacientes com esquema vacinal completo no momento da internação apresentaram uma chance 41,7% menor de morte (OR = 0,583; IC95% 0,501–0,679) por covid-19 na análise ajustada, quando comparados com pacientes não vacinados.

**CONCLUSÕES:**

O estudo revela que a imunização foi efetiva na redução da chance de óbito por covid-19. Os resultados sugerem que uma maior cobertura vacinal no primeiro semestre de 2021 evitaria milhares de mortes no país.

## INTRODUÇÃO

A covid-19 é uma doença infecciosa causada pelo novo coronavírus da síndrome respiratória aguda grave 2 (SARS-CoV-2), que surgiu no fim de 2019. Até o final do ano de 2022, a pandemia provocada pela covid-19 causou a morte de 6,67 milhões de pessoas em todo o mundo^1^. Desse total, 694 mil (10,4%) ocorreram no Brasil^2^.

No mundo, a gravidade da pandemia de covid-19 levou os países a adotarem medidas para conter o avanço da crise sanitária. Entre as estratégias adotadas, a vacinação contra a covid-19 tem sido bem-sucedida na tarefa de proteção da população e no enfrentamento coletivo da emergência em saúde^3,4^. Após o desenvolvimento de diferentes tipos de vacinas, a alta taxa de cobertura vacinal foi a mais importante estratégia para enfrentar a pandemia^5^, aliada às demais medidas não farmacológicas para o controle do contágio^6^.

Os esforços no desenvolvimento de vacinas iniciaram-se em março de 2020. Durante o primeiro ano da pandemia, diversos laboratórios divulgaram seus processos de desenvolvimento da vacina. Esses processos foram acompanhados com atenção pela comunidade internacional^9,10^. No Brasil, a vacinação da população foi iniciada em janeiro de 2021, após a aprovação dos imunizantes Coronavac (vírus vivo atenuado) da Sinovac/Butantã e o imunizante ChAdOx1 nCoV-19 (recombinante) produzido pela parceria AstraZeneca/Fiocruz. Posteriormente, a vacina da Pfizer–BioNTech (RNA Mensageiro) e o imunizante produzido pela farmacêutica Janssen (recombinante) foram incorporados ao Programa Nacional de Imunizações (PNI)^11^.

Os imunizantes utilizados no Brasil apresentam boa eficácia para a prevenção de casos moderados e graves, o que pode ser observado em ensaios clínicos^12^ e estudos de efetividade em outros países^15^. Nos estudos de efetividade, os dados apontam que os imunizantes protegem contra a evolução para formas graves, com uma redução na mortalidade por covid-19, que foi de 86,3% com o imunizante Coronavac e de 96,7% para o imunizante da Pfizer–BioNTech. Igualmente, demonstrou-se redução de pelo menos 85% nas internações por covid-19^15^.

Apesar das evidências da efetividade da vacina na população geral, pouco se sabe sobre como a situação vacinal influencia na mortalidade no subgrupo de pacientes que evoluem para formas graves da doença. Destaca-se que há poucos estudos sobre a efetividade de vacinas em países em desenvolvimento. A hipótese dos autores é que o desenvolvimento da imunidade ativa a partir de imunizantes para covid-19 confere aos indivíduos melhor capacidade de resposta imunológica durante formas graves da doença. Nesse sentido, o objetivo deste estudo foi avaliar a efetividade do esquema vacinal completo na redução da mortalidade de pessoas internadas por síndrome respiratória aguda grave (SRAG). Além disso, avaliamos os fatores de risco para a mortalidade por covid-19.

## MÉTODOS

Trata-se de estudo de coorte retrospectivo, baseado em dados extraídos do banco de dados do Sistema de Informação da Vigilância Epidemiológica da Gripe (Sivep-Gripe) e da base nacional do Programa Nacional de Imunizações (SIS-PNI), referentes ao ano de 2021, disponibilizados pela Secretaria Estadual de Saúde da Paraíba (SES-PB) e atualizados até 6 de novembro de 2021. A escolha pelo estado da Paraíba foi por conveniência, em virtude da parceria com a Universidade Federal da Paraíba (UFPB) e do acesso aos dados de internação e imunização.

O estado da Paraíba contempla 223 municípios e conta com uma alta abrangência da Atenção Básica, apresentando a quarta maior cobertura da Estratégia Saúde da Família do país em 2019 (86,7%)^18^. A relação de leitos hospitalares é de 205,3 leitos por 100 mil habitantes, sendo um pouco superior à média nacional^19^. A incidência acumulada de covid-19 no estado foi de 17.625,2 casos por 100 mil habitantes em meados de março de 2023, valor similar à média nacional. A mortalidade por covid-19, por sua vez, foi de 262,3 óbitos por 100 mil habitantes, enquanto a média nacional foi de 332,9 óbitos por 100 mil habitantes^2^.

Foram incluídas no estudo pessoas com idade ≥ 18 anos internadas com covid-19 e que iniciaram sintomas clínicos entre 1º de fevereiro de 2021 e 6 de novembro de 2021. Foram consideradas pessoas com covid-19 todas aquelas que preencheram os critérios diagnósticos do Ministério da Saúde (confirmação laboratorial, critérios clínicos epidemiológicos) classificados no Sivep-Gripe, como síndrome respiratória aguda grave por covid-19, e que estavam registradas como pessoas internadas.

Foram excluídos do estudo os casos em que o registro da internação e a evolução clínica (óbito por covid-19, óbito por outras causas ou cura) não foram registrados na base do Sivep-Gripe. Optou-se por avaliar dados referentes a um período após o início do processo de imunização com o intuito de garantir que pessoas imunizadas e não imunizadas estavam expostas a cepas virais semelhantes.

As informações de identificação do paciente, sexo, data dos primeiros sintomas, idade, presença de fatores de risco para covid-19 (presença ou ausência de qualquer fator de risco), código do estabelecimento de internação no Cadastro Nacional de Estabelecimentos (CNES), município de residência, data da internação e evolução do caso foram extraídas do banco Sivep-Gripe. Informações relativas ao tipo do imunizante, à data de aplicação da vacina, à dose da vacina e aos dados de identificação da pessoa imunizada foram extraídas do SIS-PNI. Foi feita uma limpeza inicial nos dados do SIS-PNI para padronizar os registros de um mesmo tipo de imunizante que foram registrados de diferentes formas e para remover registros duplicados de doses de vacina para o mesmo paciente. Em seguida, as bases do SIS-PNI e Sivep-Gripe foram agregadas utilizando a técnica de *record linkage* determinístico, empregando como chave o Cadastro de Pessoa Física (CPF). Nos registros em que essa informação estava ausente, foi realizado um segundo *linkage,* utilizando as variáveis nome e data de nascimento do paciente. Os processos de filtragem, limpeza e junção dos dados foram realizados utilizando a linguagem R através do pacote *tidyverse*^20^.

Após a agregação das bases de dados, o banco foi revisado manualmente para identificar notificações duplicadas e notificações em diferentes estabelecimentos durante a evolução clínica da mesma infecção por SAR-CoV-2, já que alguns pacientes foram internados em mais de um estabelecimento (transferência ou reinternação). Nesses casos, foi considerada a notificação com data de evolução mais recente. Nos casos em que a diferença entre as datas de internação foi superior a 60 dias, considerou-se uma nova infecção, não havendo exclusão do registro.

O desfecho clínico avaliado foi o óbito por covid-19 e as variáveis explicativas foram sexo, raça/cor, tipo do estabelecimento de internação (estatal ou privado), Índice de Desenvolvimento Humano Municipal (IDHM) do município de residência, idade do paciente, presença de comorbidade e situação vacinal na data do início dos primeiros sintomas. A classificação de raça/cor seguiu a categorização do Instituto Brasileiro de Geografia e Estatística (IBGE). Pessoas categorizadas como pardas e pretas foram agregadas na categoria negra. Foram considerados estabelecimentos públicos aqueles geridos pelo poder público; e como privados, estabelecimentos privados com fins lucrativos e filantrópicos, utilizando como parâmetro a classificação jurídica da base do CNES. Os municípios foram categorizados com base nas categorizações propostas pela ONU: IDH baixo inferior a 0,555; médio entre 0,555 e 0,699; alto entre 0,700 e 0,799 e muito alto para IDH maior ou igual a 0,800^21^. A situação vacinal foi definida a partir do tempo decorrido entre a data de aplicação das doses de vacinas e a data dos primeiros sintomas. Foram considerados não vacinados todos os pacientes sem dose de vacina ou com aplicação realizada 14 dias antes da data do início dos primeiros sintomas. Pacientes com intervalo de tempo superior a 14 dias entre a data da segunda dose da vacina e a data dos primeiros sintomas foram considerados completamente imunizados e aqueles em que apenas uma dose de imunizante foi aplicada pelo menos 14 dias antes dos primeiros sintomas foram considerados parcialmente imunizados.

A população do estudo foi descrita em subgrupos categorizados a partir de características referentes às variáveis explicativas, utilizando valores absolutos e a proporção de cada subgrupo em relação ao total dos indivíduos estudados. Foi realizada regressão logística e calculados valores de *odds ratio* (OR) e seus respectivos intervalos de confiança (IC95%) para cada um dos fatores analisados, utilizando o método de regressão logística bivariada e valores de OR ajustados e seus intervalos de confiança (IC95%), a partir de análise multivariável. A variável raça/cor não foi avaliada nos modelos de regressão em virtude da alta proporção de dados faltantes, especialmente nas notificações de estabelecimentos privados.

Adicionalmente, os dados de cobertura vacinal mensal da primeira e segunda dose dos imunizantes contra a covid-19 na população da Paraíba foram calculados utilizando dados do SIS-PNI e dados referentes à população paraibana por faixa etária.

Em atenção às recomendações contidas na Resolução nº 466/2012, do Conselho Nacional de Saúde, o projeto deste estudo foi submetido e aprovado pelo Comitê de Ética em Pesquisa com Seres Humanos, do Centro de Ciências Médicas da UFPB, sob CAAE nº 50833321.4.0000.8069.

## RESULTADOS

No período estudado, houve 15.738 notificações de casos de SRAG atribuídas à infecção causada por SAR-CoV-2. A limpeza do banco de dados e a análise referente ao preenchimento dos critérios de inclusão resultaram em uma base de dados com 13.300 internações (Figura 1).

**Figura 1:** Fluxograma de inclusão de participantes a partir das notificações de SRAG segundo critérios de inclusão e exclusão do estudo. SRAG: síndrome respiratória aguda grave.

Das internações analisadas, 76% ocorreram entre os meses de fevereiro e maio de 2021. A maioria das pessoas internadas eram homens (55,95%), apresentavam comorbidades (58,27%), eram pessoas negras (79,19%) e foram internados em hospitais públicos (80,35%) (Tabela 1). A variável raça/cor apresentou o pior preenchimento entre as variáveis avaliadas. O percentual de pacientes sem registro de raça/cor nas notificações foi de 2,40% em hospitais públicos e de 55,34% em hospitais privados. O percentual de pacientes negros internados em hospitais variou entre 52,64% nos hospitais privados e 93,04% em hospitais públicos. Entre os pacientes que foram a óbito, 74,26% obtiveram acesso a Unidades de Terapia Intensiva (UTI) em hospitais públicos e 84,78% em hospitais privados. A maior parte dos pacientes internados tinha idade ≤ 60 anos (59,64%) e a média de idade foi de 56,64 anos. A letalidade dos casos de SRAG por covid-19 em pacientes internados foi de 36,22%, variando por faixa etária, sendo de 19,13% em pacientes com até 39 anos e de 60,73% em pacientes com idade ≥ 80 anos. A letalidade variou ao longo do tempo, sendo de 41,1% nos meses de fevereiro e março e de 29,6% nos meses de junho e julho.

**Tabela 1:** Característica de pacientes internados por SRAG no estado da Paraíba, segundo situação vacinal antes da infecção por covid-19.

Característica	Situação vacinal anterior à data dos primeiros sintomas							
	Esquema completo		Esquema incompleto		Sem vacina		Total	
	n	(%)	n	(%)	n	(%)	n	(%)
Sexo								
Masculino	495	49,25	738	52,05	6.209	57,08	7.442	55,95
Feminino	510	50,75	680	47,95	4.668	42,92	5.858	44,05
Raça/cor								
Brancos	110	10,17	122	11,28	850	78,56	1.082	8,14
Negros	770	7,31	1.128	10,71	8.634	81,89	10.532	79,19
Amarelos	10	7,81	9	7,03	109	85,16	128	0,96
Indígenas	3	20	2	13,33	10	66,67	15	0,11
Sem informação	112	7,26	157	10,17	1.274	82,57	1.543	11,6
Presença de fator de risco								
Não	235	23,38	381	26,87	4.934	45,36	5.550	41,73
Sim	770	76,62	1.037	73,13	5.943	54,64	7.750	58,27
Faixa etária (anos)								
18–39	18	1,79	59	4,16	2.427	22,31	2.504	18,83
40–49	17	1,69	65	4,58	2.469	22,7	2.551	19,18
50–59	29	2,89	115	8,11	2.733	25,13	2.877	21,63
60–69	133	13,23	420	29,62	1.557	14,31	2.110	15,86
70–79	388	36,61	334	23,55	1.054	9,69	1.776	13,35
≥ 80	420	41,79	425	29,97	637	5,86	1.482	11,14
Tipo de estabelecimento de internação								
Público	825	7,72	1.143	10,7	8.719	81,59	10.687	80,35
Privado	151	6,72	238	10,6	1.857	82,68	2.246	16,89
Sem informação	29	7,9	37	10,08	301	82,02	367	2,76
IDH do município de residência								
Baixo	44	9,61	61	13,32	353	77,07	458	3,44
Médio	565	9,02	654	10,44	5.048	80,55	6.267	47,12
Alto	395	6,04	698	10,67	5.451	83,3	6.544	49,2
Muito alto	1	3,23	55	16,13	25	80,6	81	0,23
Tipo de imunizante								
Sinovac/Butantã	936	7,04	774	5,82	-	-	1.710	12,86
Oxford/AstraZeneca/Fiocruz	69	0,52	588	4,42	-	-	657	4,94
Janssen Cilag	-	-	7		-	-	7	0,05
Pfizer BioNTech	-	-	48		-	-	48	0,36

IDH: Índice de Desenvolvimento Humano.

Conforme se observa na Tabela 1, o grupo de pessoas internadas com esquema vacinal completo apresentou características distintas do grupo sem imunização prévia à internação por SRAG, sendo predominantemente composto por idosos (93,63%) e por pessoas portadoras de fator de risco para óbito por covid-19 (76,62%). O percentual de indígenas com esquema completo foi superior ao dos demais grupos, em decorrência de ter sido um grupo prioritário pelo PNI. Os pacientes internados com esquema vacinal completo utilizaram os imunizantes da Sinovac/Butantã (93,13%) e Oxford/AstraZeneca/Fiocruz (6,87%). Esses imunizantes também foram os mais usados pelos pacientes com esquema vacinal incompleto. O percentual de pacientes com esquema vacinal completo foi similar entre os pacientes hospitalizados em hospitais públicos e privados. Entre as pessoas internadas, a mediana do tempo decorrido entre a segunda dose da vacina e a data dos primeiros sintomas foi de 79 dias (intervalo interquartílico de 46 a 124 dias). Destaca-se que o maior percentual de pacientes internados com esquema vacinal completo ocorreu em municípios com IDHM baixo e médio. Cardiopatia, diabetes e obesidade foram os fatores de risco mais identificados nos pacientes internados (Tabela 2).

**Tabela 2:** Prevalência de fatores de risco em pacientes hospitalizados.

Fator de risco	Prevalência (%)
Cardiopatia	27,36
Diabetes	9,76
Obesidade	11,98
Imunossupressão	1,45
Pneumopatia	1,91
Asma	1,86
Hepatopatia	0,56
Renal	2,37
Puerpério	0,5
Síndrome de Down	0,44
Hematológica	0,34
Outras comorbidades	34,39

A cobertura vacinal na Paraíba apresentou variações por faixas etárias, com menor abrangência para as pessoas mais jovens (Figura 2). A população com mais de 60 anos teve cobertura maior que 90% na primeira dose a partir do mês de abril. O alcance da segunda dose foi inferior ao observado na primeira, com percentuais acima de 90% identificados apenas na população com 70 anos ou mais.

**Figura 2:** Cobertura vacinal da primeira dose (2A) e segunda dose (2B) da vacina contra covid-19 na população de adultos e idosos no estado da Paraíba, por faixa etária.

Os dados da análise multivariável, descritos na Figura 3, demonstram que mulheres têm menor chance de óbito do que homens (OR = 0,896; IC95% 0,830–0,967). Por outro lado, pacientes com algum fator de risco apresentam chance de óbito 62,7% maior que pacientes sem fatores de risco (OR = 1,627; IC95% 1,500–1,765). A idade foi outro aspecto associado com o aumento progressivo do risco de óbito. A população com idade ≥ 80 anos teve chance de óbito (OR = 7,426; IC95% 6,309–8,741) maior que a população entre 18 e 39 anos. Pacientes com esquema vacinal completo apresentaram uma chance 41,7% menor de morte (OR = 0,583; IC95% 0,501–0,679) por covid-19, quando comparados com pacientes não vacinados. Indivíduos com esquema vacinal incompleto demonstraram redução da chance de óbito (OR = 0,699; IC95% 0,615–0,795), mas inferior ao subgrupo com esquema completo. Pessoas internadas em hospitais privados tiveram menor chance de óbito quando comparadas a pessoas internadas em hospitais públicos (OR = 0,756; IC95% 0,679–0,842).

**Figura 3:** *Odds ratio* da evolução para óbito em pacientes internados com síndrome respiratória aguda grave. Análises bivariada (Figura 3A) e multivariável (Figura 3B), considerando características biológicas, situação vacinal e características assistenciais e socioeconômicas.

## DISCUSSÃO

Os resultados apontam que as vacinas foram efetivas na redução da chance de óbito por covid-19 em pessoas com formas graves da doença. Algumas características estiveram associadas ao óbito por covid-19, tais como: sexo masculino, idade ≥ 40 anos, presença de comorbidades e condições de saúde identificadas como fatores de risco no Sivep-gripe e internação em hospitais públicos.

A maioria das internações analisadas ocorreu entre fevereiro e maio de 2021, período em que havia escassez de imunizantes no país. No fim de maio de 2021, a cobertura vacinal da segunda dose na Paraíba em pessoas com mais de 18 anos era de apenas 13,44%^22^. Apesar do histórico de campanhas de vacinação bem-sucedidas e uma boa taxa de aceitação da vacina contra covid-19, o Brasil apresentou um baixo aumento de cobertura vacinal no primeiro semestre de 2021^23^. O atraso na aquisição de imunizantes, em virtude de negativas a propostas da Pfizer em 2020, fez com que o país iniciasse a imunização quase um mês depois de países como o Canadá^23^. O menor estoque dos imunizantes também gerou interrupções parciais do processo de imunização. Um estudo utilizando a modelagem SIR (suscetível-infectado-recuperado) aponta que a antecipação do início da vacina e um ritmo maior de vacinação nos meses iniciais poderiam ter evitado milhares de mortes^23^.

Um estudo de coorte, que também utilizou o banco do Sivep-Gripe, analisou 254 mil internações no Brasil em 2020 e apontou que 56% dos pacientes internados eram do sexo masculino, 53% tinham idade ≥ 60 anos e 84% apresentavam, pelo menos, uma comorbidade^24^. Na região Nordeste, os percentuais foram, respectivamente, de 58%, 58% e 83%^24^.

O alto percentual de internações em hospitais públicos revela a importância do Sistema Único de Saúde (SUS) na resposta à epidemia de covid-19. Os dados dos pacientes internados na Paraíba entre fevereiro e novembro de 2021 demonstram um percentual menor de pessoas internadas com idade ≥ 60 anos (40,4%) e com comorbidades (58,27%) quando comparados com dados do Sivep-gripe do país e da região Nordeste em 2020^24^. Esses grupos populacionais iniciaram o processo de imunização de forma prioritária e apresentaram um maior percentual de indivíduos com esquema vacinal completo quando comparados com pacientes mais jovens ou sem comorbidades. Assim, essa redução no percentual de internados indica o impacto da vacinação na redução de formas graves. A letalidade encontrada na Paraíba em 2021 (36,22%) foi inferior à observada na região Nordeste em 2020 (48%)^24^. De forma similar, a ampliação da cobertura vacinal entre os idosos brasileiros até meados de maio de 2021 foi associada a declínios na letalidade relativa em comparação com indivíduos mais jovens^3^.

A maior parte dos pacientes internados por SRAG durante o período do estudo apresentou fatores de risco e idade superior a 50 anos, o que está em consonância com outros estudos^25,26^. O maior percentual de pessoas negras internadas foi similar ao valor encontrado para o Nordeste em estudo anterior^24^. Entretanto, esse percentual é superior ao esperado, já que a população negra no estado da Paraíba representava 67,1% do total em 2021^27^. Esse fato pode indicar que as pessoas negras estavam mais expostas à contaminação por covid-19, conforme outros estudos da literatura ^28,29^. Outro fator que pode ter contribuído para essa diferença foi a taxa de não preenchimento dessa variável na rede privada, em que o percentual de pacientes brancos internados era superior, gerando, assim, um subdimensionamento. Esse alto percentual de não preenchimento aponta a importância de fortalecer o processo de qualificação das equipes e do preenchimento dessas informações nos estabelecimentos privados.

Outro ponto de destaque é uma chance de morte 24,40% menor em pessoas internadas em hospitais privados. Uma das hipóteses para a maior mortalidade nos hospitais públicos é a falta de acesso a cuidados intensivos. O percentual de pessoas que foram a óbito sem acesso à UTI em hospitais públicos (25,7%) foi 69% maior do que o encontrado em hospitais privados (15,20%). Estudos internacionais apontam que desigualdades endêmicas na carga de doenças crônicas em pessoas com vulnerabilidades socioeconômicas podem contribuir para uma maior mortalidade^28,29^. Assim, o impacto da determinação social das doenças reflete desigualdades preexistentes, resultando em uma sindemia de covid-19^30^. Em uma perspectiva oposta, a similaridade entre os percentuais de pacientes com esquema completo internados em hospitais públicos e privados e um maior percentual de pessoas com duas doses em municípios com IDHM baixo e médio entre as pessoas hospitalizadas revela que o PNI permitiu acesso à vacina a pessoas de todas as classes sociais e municípios de todos os graus de desenvolvimento humano, atuando como um produtor de equidade.

Os dados indicam que a imunização contra a covid-19 atua como fator de proteção em pacientes hospitalizados. Um ensaio clínico que avaliou o uso de dexametasona em pacientes hospitalizados por covid-19 demonstrou uma redução relativa de risco de 17%^31^. Dois ensaios clínicos em pacientes internados não demonstraram redução da mortalidade com o remdesivir^32,33^. A redução da letalidade identificada nesta coorte retrospectiva não pode ser diretamente comparada aos ensaios clínicos supracitados; entretanto, sugere que o papel dos imunizantes na proteção de pacientes com formas graves de covid-19 apresenta relevância clínica.

Este estudo tem limitações relacionadas ao uso de bases de dados secundárias. A primeira das limitações refere-se a pacientes dos quais não houve o registro da classificação diagnóstica. Esses casos podem refletir uma subnotificação no número de pessoas internadas por covid-19. Outra limitação refere-se a casos de pacientes com covid-19 cujo óbito foi atribuído a outra causa (25 pacientes). Apesar das estratégias de *linkage* utilizadas, a identificação da situação vacinal dos pacientes internados pode ter sido prejudicada em razão de dados faltantes nos bancos de dados utilizados ou erros de preenchimento. Destaca-se como limitação o percentual de dados sobre raça/cor em mais da metade das notificações ocorridas em hospitais privados. Os achados referem-se a um momento específico da epidemia em que apenas uma parcela da população estava vacinada. Mais de 93% das pessoas que realizaram esquema completo utilizaram a vacina da Sinovac/Butantã. Assim, os resultados encontrados se relacionam majoritariamente ao uso desse imunizante. A Sinovac/Butantã apresenta a menor efetividade na redução de hospitalizações e mortes entre os imunizantes utilizados no Brasil^34^. Outras vacinas podem gerar maior redução da mortalidade. Dessa forma, sugere-se a realização de novos estudos para avaliar a redução da chance de óbitos de todos os imunizantes.

Outras pesquisas evidenciaram a importância dos imunizantes para a redução das formas graves e da mortalidade para a população geral. Este estudo conclui que pessoas hospitalizadas com vacinação completa apresentam menor letalidade quando comparadas às pessoas que não tinham o esquema completo antes da internação, sendo mais uma evidência da importância das vacinas contra a covid-19. Além da redução da morbidade e mortalidade, o PNI demonstrou, novamente, sua importância como um indutor de equidade. Tem-se o grande desafio de ampliar a cobertura vacinal em grupos que se tornaram refratários ao uso de imunizantes e que ainda estão vulneráveis à infecção pelo SARS-CoV-2. O fortalecimento do SUS e do PNI também será necessário para reverter as baixas coberturas vacinais de vários imunizantes.
